# Supplementary material for: Evaluation of Quality, Antioxidant Capacity, and Digestibility of Chickpea (Cicer arietinum L. cv Blanoro) Stored under N2 and CO2 Atmospheres
Source: Molecules. 2021 May 8;26(9):2773. doi: 10.3390/molecules26092773 (PMC8125957; doi:10.3390/molecules26092773)
Supplement: Supplementary file 1 [file molecules-26-02773-s001.zip › Figures S1-S4.pdf]

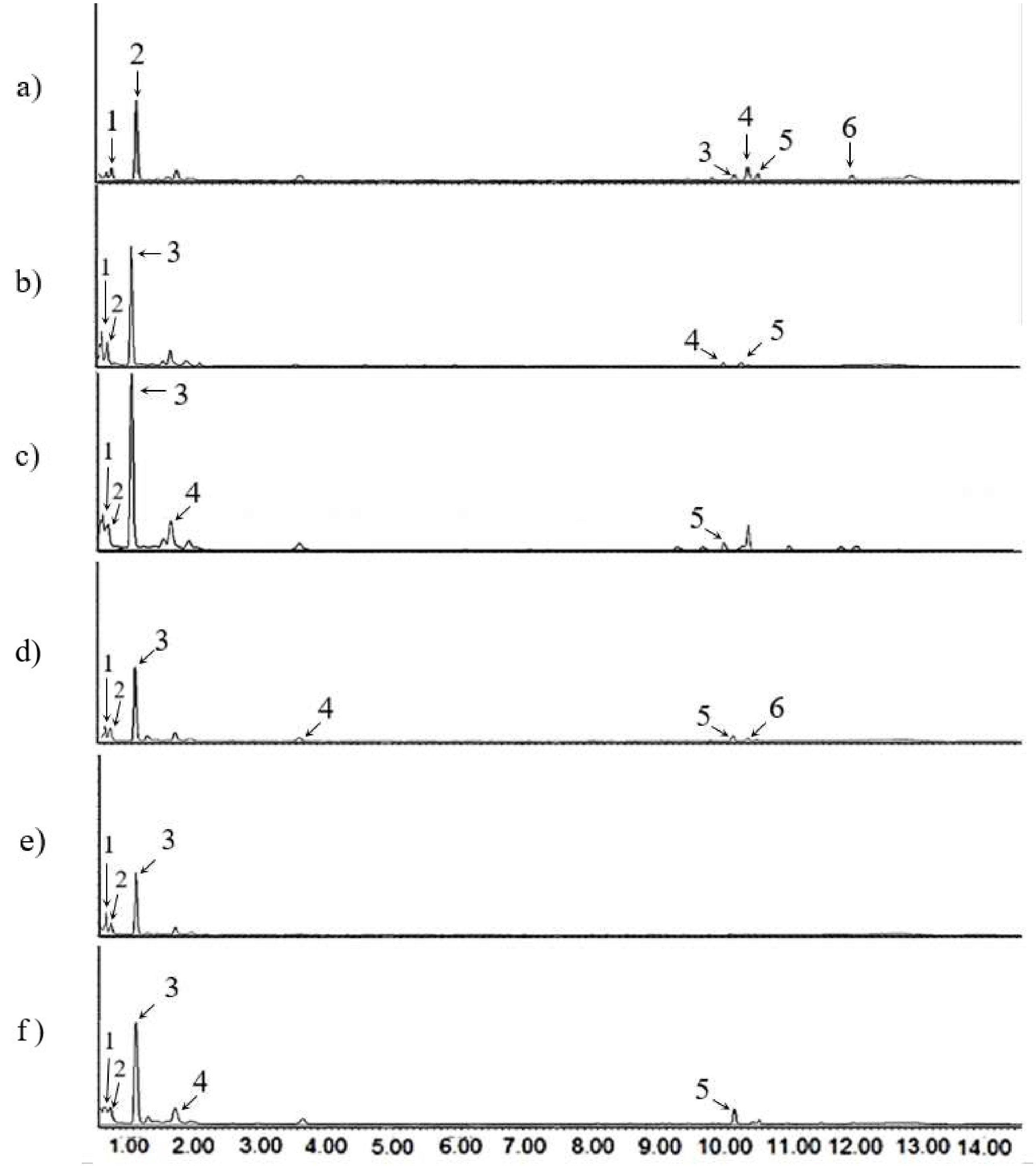

Figure S1. UPLC-MS chromatograms of the phenolic compounds identified in raw and cooked chickpea. (**a**) Raw chickpea Free phenolic fraction (1 = Gomisin D, 2 = Enterodiol, 3 = Hydroxytyrosol 4-*O*-glucoside, 4 = Pelargonidin 3,5-*O*-diglucoside, 5 = 1,4-Naphtoquinone, 6 = *p*-Coumaroyl glucose); (**b**) Raw chickpea conjugated phenolic fraction (1 = 6-Geranylnaringenin, 2 = Gomisin D, 3 = Enterodiol, 4 = Isorhamnetin, 5 = Pelargonidin 3,5-*O*-diglucoside); (**c**) Raw chickpea bound phenolic fraction (1 = Hydroxytyrosol 4-*O*-glucoside, 2 = Gomisin D, 3 = Enterodiol, 4 = Anhydrosecoisolariciresinol, 5 = Phloretin 2′-*O*-glucoside); (**d**) Cooked chickpea free phenolic fraction (1 = Hydroxytyrosol 4-*O*-glucoside, 2 = Gomisin D, 3 = Enterodiol, 4 = Hespertin 3′,7-*O*-diglucuronide, 5 = Hydroxytyrosol 4-*O*-glucoside, 6 = Pelargonidin 3,5-*O*-diglucoside); (**e**) Cooked chickpea conjugated phenolic fraction (1 = 6-Geranylnaringenin, 2 = Gomisin D, 3 = Enterodiol); (**f**) Cooked chickpea bound phenolic fraction (1 = Hydroxytyrosol 4-*O*-glucoside, 2 = Gomisin D, 3 = Enterodiol, 4 = Anhydrosecoisolariciresinol, 5 = 3,4-Diferuloylquinic acid).

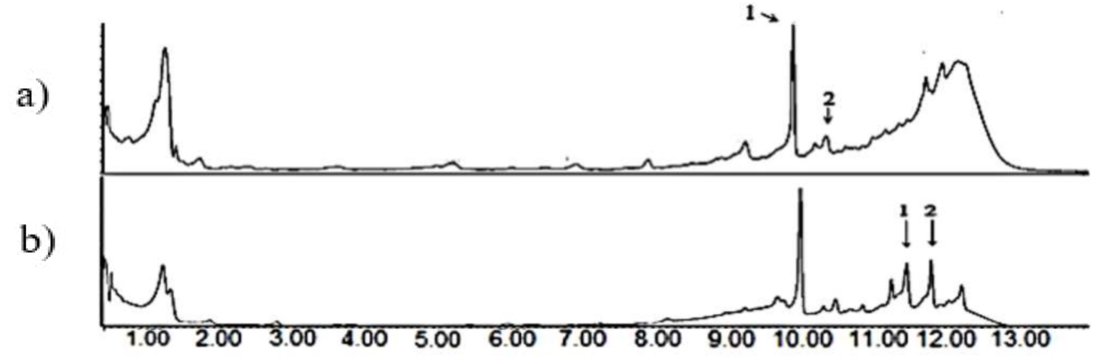

Figure S2. UPLC-MS chromatograms of the phenolic compounds identified in the mouth. (**a**) Free phenolic fraction (1 = Caffeoyl tartaric acid, 2 = *p*-Coumaroyl tyrosine); (**b**) Bound phenolic fraction (1 = Syringaldehyde, 2 = Glycitein)

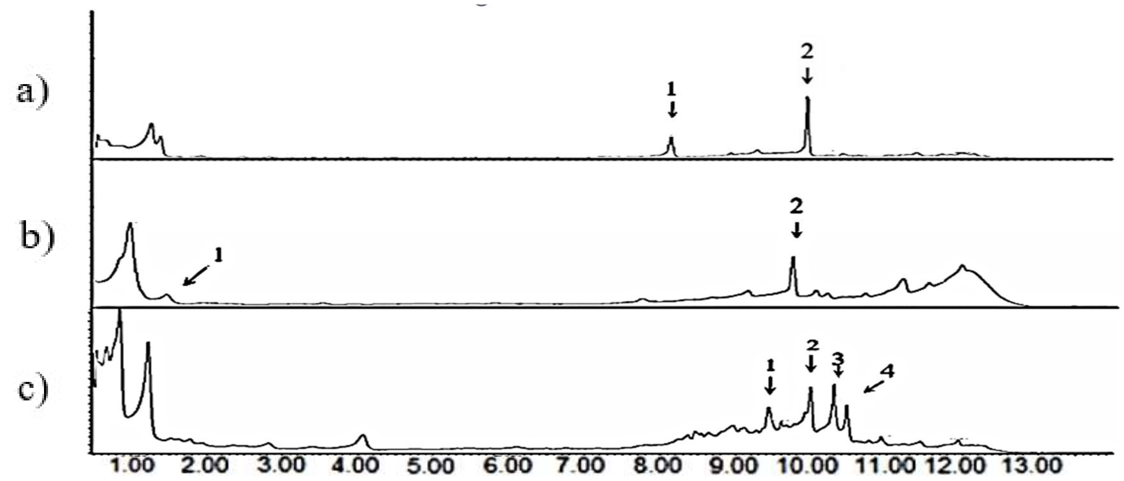

Figure S3. UPLC-MS chromatograms of the phenolic compounds identified in the stomach. (**a**) Free phenolic fraction (1 = Kaempferol 3′-*O*-(2″-rhamnosyl-galactoside)7-*O*-rhamnoside, 2 = Delphinidin 3-O-(6''-acetyl-glucoside)); (**b**) Conjugated phenolic fraction (1 = 6-Geranylnaringenin, 2 = 1-Caffeoyl-5-feruloylquinic acid); (**c**) Bound phenolic fraction (1 = (-)-Epigallocatechin 3-*O*-glucoronide, 2 = Delphinidin 3-*O*-(6″-acetyl-glucoside)).

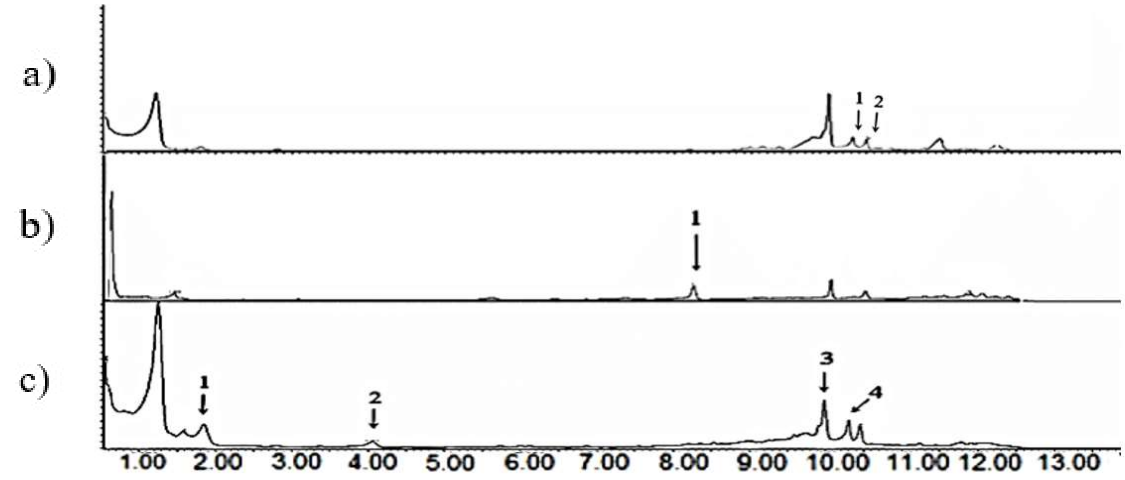

Figure S4. UPLC-MS chromatograms of the phenolic compounds identified in the intestine. (**a**) Free phenolic fraction (1 = Tyrosol 4-sulfate, 2 = (-)-Epicatechin-(2a-7)(4a-8)-epicatechin 3-O-galactoside); (**b**) Conjugated phenolic fraction (1 = Pelargonidin 3-O-glucosyl-rutinoside); (**c**) Bound phenolic fraction (1 = Caffeoyl glucosa, 2 = Pseudobaptigenin, 3 = Delphinidin 3-*O*-(6″-acetyl-glucoside), 4 = Tyrosol 4-sulfate).
