# Supplementary material for: Evaluation of Quality, Antioxidant Capacity, and Digestibility of Chickpea (Cicer arietinum L. cv Blanoro) Stored under N2 and CO2 Atmospheres
Source: Molecules. 2021 May 8;26(9):2773. doi: 10.3390/molecules26092773 (PMC8125957; doi:10.3390/molecules26092773)
Supplement: Supplementary file 1 [file molecules-26-02773-s001.zip › Table S1.pdf]

Table S1. Pearson´s correlation coefficients of Total phenolic compounds versus raw and cooked chickpea, with the different antioxidant techniques (ABTS, DPPH, FRAP, AAPH).

		Free			Conjugate			Bound		
		**r**	**R^2**	**Regression equation**	**r**	**R^2**	**Regression equation**	**r**	**R^2**	**Regression equation**
**Raw chickpea**	ABTS	**0.911**	0.831	y = 0.2487x + 1.3084	**0.952**	0.908	y = 3.0772x - 64.108	**0.887***	0.787	y = 0.2975x - 1.105
	DPPH	**0.867**	0.752	y = 0.2137x + 4.5855	**0.982**	0.965	y = 0.5194x + 3.183	**0.885***	0.784	y = 0.536x - 43.363
	FRAP	**0.449***	0.202	y = 0.021x + 17.587	**0.814***	0.663	y = 0.1063x + 63.468	**0.926***	0.858	y = 0.0273x + 67.30
	AAPH	**0.911***	0.830	y = 0.1538x + 2.6971	**0.879***	0.773	y = 1.7113x + 22.097	**0.950***	0.902	y = 0.2286x + 43.08
**Cooked chickpea**	ABTS	**0.909**	0.826	y = 0.1093x + 6.1471	**0.852**	0.877	y = 0.1776x + 5.6806	**0.938***	0.880	y = 0.0712x + 13.89
	DPPH	**0.708**	0.502	y = 0.26x + 1.8703	**0.950**	0.903	y = 1.2526x - 14.664	**0.945***	0.893	y = 0.1263x + 13.37
	FRAP	**0.975***	0.464	y = 0.878x + 2.7693	**0.928***	0.903	y = 1.2526x - 14.664	**0.966***	0.933	y = 0.0264x + 27.64
	AAPH	**0.941***	0.885	y = 0.6273x - 7.5899	**0.873***	0.762	y = 1.477x + 3.076	**0.927***	0.792	y = 0.0617x + 36.06

*Correlation is significant at $p < 0.01$.
